# Choline Chloride–Lactic Acid-Based NADES As an Extraction Medium in a Response Surface Methodology-Optimized Method for the Extraction of Phenolic Compounds from Hazelnut Skin

**DOI:** 10.3390/molecules26092652

**Published:** 2021-05-01

**Authors:** Chiara Fanali, Valeria Gallo, Susanna Della Posta, Laura Dugo, Leone Mazzeo, Marco Cocchi, Vincenzo Piemonte, Laura De Gara

**Affiliations:** 1 Department of Science and Technology for Humans and the Environment, Campus Bio-Medico University of Rome, Via Álvaro del Portillo 21, 00128 Rome, Italy; v.gallo@unicampus.it (V.G.); s.dellaposta@unicampus.it (S.D.P.); l.dugo@unicampus.it (L.D.); l.degara@unicampus.it (L.D.G.); 2 Department of Engineering, Campus Bio-Medico University of Rome, Via Álvaro del Portillo 21, 00128 Rome, Italy; leone.mazzeo@uniroma1.it (L.M.); m.cocchi@unicampus.it (M.C.); v.piemonte@unicampus.it (V.P.); 3 Department of Chemical Engineering Materials & Environment, Sapienza University of Rome, Via Eudossiana 18, 00184 Rome, Italy

**Keywords:** hazelnut skin, deep eutectic solvents, phenolic compounds, response surface methodology, ultrasound-assisted solid-liquid extraction

## Abstract

Deep eutectic solvents (DESs) are promising green solvents for the extraction of compounds from food byproducts. Hazelnut (*Corylus avellana L*.) is one of the most commonly cultivated tree nuts worldwide. The skin represents one of the major byproducts of the hazelnut industry and accounts for 2.5% of the total hazelnut kernel weight. It is a rich source of phenolic compounds like flavan-3-ols, flavonols, dihydrochalcones, and phenolic acids. In this work, fifteen DESs based on choline chloride and betaine, with different compositions, were studied in order to test their phenolic compounds extraction efficiency through the determination of their total concentration via Folin–Ciocalteu assay. A qualitative analysis of extracted phenolic compounds was assessed by HPLC with UV and MS detection. Using the DES with the best extraction efficiency, a new ultrasound-assisted solid liquid extraction (UA-SLE) method was optimized though the response surface methodology (RSM), taking into account some extraction parameters. Efficient recovery of extracted phenolic compounds was achieved using a 35% water solution of choline chloride and lactic acid (molar ratio 1:2) as an extraction solvent, working at 80 °C and with a solid-to-solvent ratio of 1:25 gmL^−1^. The optimized conditions made it possible to recover 39% more phenolic compounds compared to a classic organic solvent.

## 1. Introduction

A wide range of dry fruits from deciduous trees, such as almonds, nuts, cashew nuts, hazelnuts, macadamias, pine nuts, pistachios, and walnuts, have been consumed since ancient times as an important source of energy [1]. The Food and Drug Administration (FDA) and European Food Safety Authority (EFSA) recommend a daily consumption of nuts, including hazelnut, for coronary heart disease risk reduction (42.5 g according to the FDA and 30 g according to the EFSA) [2]. Hazelnut (*Corylus avellana L.*, *Betulaceae* family) is one of the most commonly cultivated tree nuts worldwide [3]. World hazelnut production was around 1.1 million tons/year in 2019 [4]. Turkey is the leading producer, accounting for about 70% of total supply, and Italy is the second largest hazelnut-producing area [5]. Hazelnut is characterized by the presence of phenolic compounds such as flavan-3-ols, benzoic acids, and flavonols [5]. During processing of hazelnut, a large number of waste products, such as hazelnut skin and other byproducts, including hard shell, green leafy cover, and tree leaf, are generated [6]. The skin represents about 2.5% of the total hazelnut kernel weight and is usually removed by blanching or roasting in order to improve the kernel flavor, color, and crunch and for the use of the kernel in the confectionery industry and bakeries. Hazelnut skins are used without treatment for animal feed, but several studies have shown the possibility of using hazelnut skins as an inexpensive source of bioactive compounds to apply in nutraceutical, pharmaceutical, or cosmetic products [3]. Hazelnut skins are a rich source of phenolic compounds. According to studies from the literature, the main phenolic subclass is by far the flavan-3-ols, in both their monomeric and polymeric forms, accounting for more than 95% of the total hazelnut skin phenolic compounds. Flavonols and dihydrochalcones represent an additional 3.5% while phenolic acids account for less than 1% of the total identified phenolics. Proanthocyanidins (PAs) are oligomers or polymers, also known as condensed tannins, classified as procyanidins, propelargonidins, or prodelphinidins on the basis of the flavan-3-ol units (epi)catechin, (epi)afzelechin, or (epi)gallocatechin, respectively [1,7]. Phenolic compounds from hazelnut skin are typically extracted using a solid-liquid extraction technique with organic solvents such as methanol, ethanol, and methanol/water mixtures [1,3]. With the emergence of green chemistry, interest in the search for green solvents has grown. Ethanol or water/ethanol mixtures have been used as green conventional solvent for the extraction of phenolic compounds in some food and food byproducts; for example, in chickpeas [8] and grape peels, seeds, and stems [9]. Alternatively, deep eutectic solvents (DESs), which were first identified by Abbott and colleagues [10], have aroused considerable attention due to their remarkable physicochemical properties, such as non-volatility, thermal stability, conductivity, and excellent solubility [11]. DESs are inexpensive, more synthetically accessible, and biodegradable and can be employed in fields relating to electrochemistry, organic reactions, catalysis, nanomaterials, extraction, and separation thanks to their green and excellent physicochemical properties [11]. DESs are mainly mixtures consisting of a hydrogen bond donor (HBD) and a hydrogen bond acceptor (HBA). By simply mixing the HBA component (most often a quaternary ammonium salt or metal salt) with the HBD component (various alcohols, amides, amines, or acids) in a given molar ratio, a homogeneous solvent is usually formed with a melting point lower than the melting points of the pure constituents. When each component that composes the DES is natural, we can define it as a natural deep eutectic solvent (NADES) [12]. Some points regarding the main chemical and physical characteristics of DES can be noted in relation to the nature of HBAs and HBDs. In general, hydrophilic DESs are more viscous when compared to ionic and non-ionic hydrophobic DESs. Furthermore, it has been noted that the viscosity of hydrophilic DESs is influenced more by HBDs. Regarding density, similar points can be made: hydrophobic DESs have densities close to or lower than the density of water while hydrophilic DESs show higher densities [13]. In general, DESs and NADESs are less toxic than most organic solvents, and NADESs are less toxic than DESs. Hayyan et al. conducted a study showing the relationship between the toxicity of NADESs, based on choline chloride, and HBDs [14]. In addition, predictive multitasking quantitative structure–toxicity relationship (mtk-QSTR) in silico studies have provided a ranking of some HBDs in terms of their contribution to the toxicity of the final DES. Among the various HBDs, alcoholic sugars and linear chain alcohols showed a negative contribution to the final solvent toxicity, while sugars and amides provided a medium level of toxicity and, with the exception of lactic acid, the organic and inorganic acids tested had the highest contributions to the final toxicity [15].

Some studies have been published on the extraction of phenolic compounds from both waste and food matrices using DESs, which showed good extraction capacities compared to traditional solvents [16]. Meng et al. evaluated different ChCl-based DESs for the extraction of quercetin, kaempferol, naringenin, and isoramnetin from *Pollen Typhae* samples [17]. Lakka et al. described the extraction of anthocyanins and flavonols from saffron using a DES consisting of lactic acid and glycine in a 5:1 molar ratio [18]. UA extraction of phenolic compounds from olive oil using several DESs and NADESs with ChCl and betaine has been reported [19]. In 2016, Khezeli et al. reported the extraction of phenolic compounds from almond, demonstrating that DESs prepared with choline chloride (ChCl) combined with ethylene glycol and/or glycerol were better than ethylene glycol or glycerol alone, probably due to the higher degree of hydrogen bonding and the greater number of electrostatic interactions in DESs with target analytes compared to pure ethylene glycol and glycerol [20]. Among the methods developed, some involved food waste matrices. Ozturk et al. conducted a study on orange peel waste samples, testing various DESs composed of ChCl as the HBA and ethylene glycol and glycerol as the HBD, in various molar ratios [21]. DESs composed by ChCl and betaine as the HBA were also used for the extraction of bioactive compounds from spent coffee grounds, obtaining good results in terms of efficiency [22]. Bonacci et al. tested some NADESs using a microwave-assisted extraction (MAE) approach to obtain phenolic compounds from olive oil processing waste, proving that this is an effective alternative extraction media [23].

To the best of our knowledge, there are no studies in the literature that have tested DESs for the extraction of bioactive molecules from hazelnut skin. The aim of this study was the development of an ecofriendly method for the extraction of bioactive phenolic compounds from hazelnut skin based on solid-liquid extraction using DESs. Obtained extracts from hazelnut skins, using betaine and ChCl-based DESs, were characterized in terms of phenolic compounds content using high performance liquid chromatography (HPLC) coupled with a photodiode array (PDA) and mass spectrometry (MS) detector method. Among the tested DESs, one was selected as the best for the extraction of phenolic compounds from the hazelnut skin. For the optimization of the extraction method, a response surface methodology (RSM) was implemented, considering as independent variables the quantity of water to be added to the DES, the solid-to-solvent ratio, and the temperature of the extraction. The results, obtained using optimized conditions, were compared with those obtained with the most commonly used ethanolic UAE method.

## 2. Results and Discussion

### 2.1. Screening of DESs

The structure of DESs determines their chemical and physical properties and, consequently, influences the extraction efficiency of targeted compounds.

Eight different NADESs were tested, using choline chloride and betaine as the HBA in combination with: lactic acid (NADES-2; NADES-14), glucose (NADES-3), xylitol (NADES-4), glycerol (NADES-5, NADES-11), and malic acid (NADES-10, NADES-15). Seven different DESs were tested, based on the same HBA, in combination with: urea (DES-1), 1,6-hexanediol (DES-6), triethylene glycol (DES-7, DES 13), ethylene glycol (DES-8, DES-12), and propylene glycol (DES-9) (Table 1).

**Figure 1:**
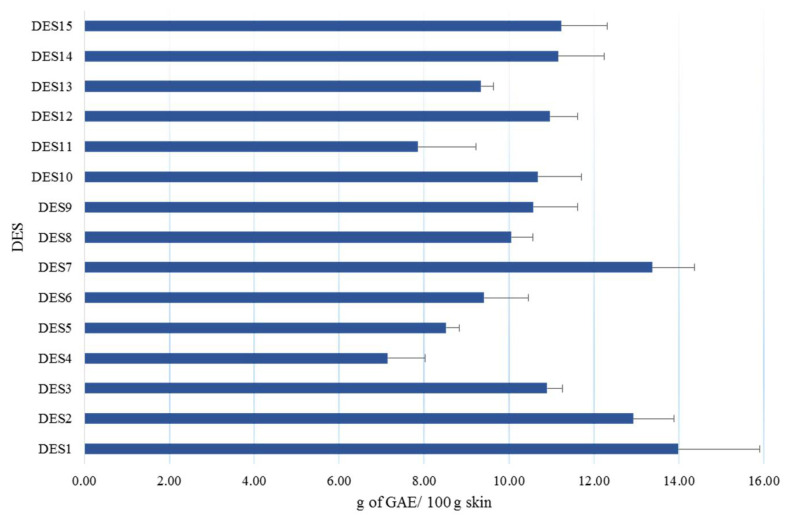
Effect of DES composition (Table 1) on the extraction efficiency of phenolic compounds.

The selection of DES-based extraction parameters was undertaken by determining the total phenolic compound concentration, expressed as grams of gallic acid equivalent (g GAE) per 100 g^−1^ of skin as a variable. In order to compare the selected DESs, all extractions were carried out under the same conditions by weighing 0.5 g of hazelnut skin and using a volume of 5 mL of extraction solvent at a temperature of 70 °C, as described in Section 3.7. Figure 1 shows the total concentration of extracted phenolic compounds with the different solvents. In general, the obtained results showed some differences in the extractability of phenolic compounds with tested DESs. No significant trend was noted in the extractions that could lead to a better performance with regard to the HBA or HBD components. The lowest amounts of total phenolic compounds were obtained using NADES-4 (7.14 g GAE 100 g^−1^ of skin) and NADES-11 (7.85 g GAE 100 g^−1^ of skin), while the highest was obtained by using DES-1, NADES-2, and DES-7, which resulted in 13.98 g GAE 100 g^−1^ of skin, 12.92 g GAE 100 g^−1^ of skin, and 13.37 g GAE 100 g^−1^ of skin, respectively. Furthermore, the ANOVA test carried out showed that there was no statistical significance among the results obtained for NADES-3, NADES-14, DES-12, or NADES-15.

Among the DESs showing the highest extraction yields, DES-1 (ChCl and urea) showed an unsatisfactory repeatability, with intra- and inter-day values higher than 14%, and for this reason it was discarded. In addition, it was also the extraction solvent that showed the worst repeatability in the extraction of phenolic compounds from olive oils [19]. Considering extraction yield and solvent toxicity, the NADES with ChCl and lactic acid molar ratio of 1:2 (NADES-2) was used as the extraction solvent for further optimization.

### 2.2. Characterization of ChCl–Lactic Acid Molar Ratio 1:2-Based DES

#### 2.2.1. FTIR Spectrum

**Figure 2:**
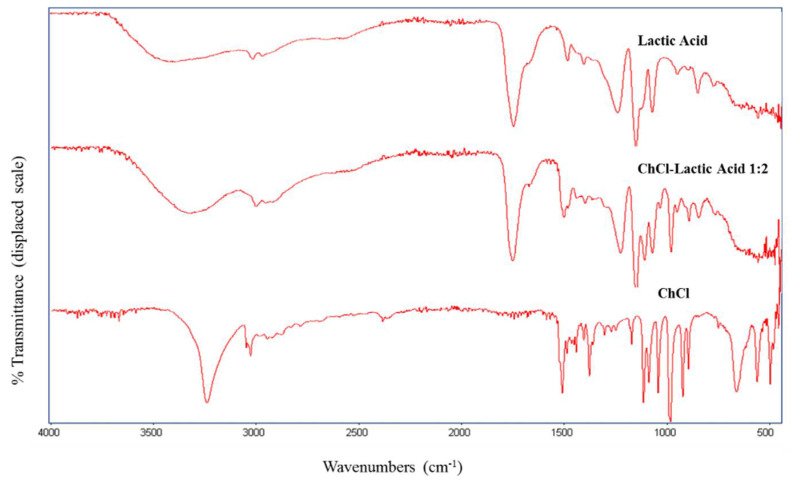
FTIR spectra of lactic acid, ChCl–lactic acid molar ratio 1:2, and ChCl.

Figure 2 shows the spectra of the HBA, HBD, and formed NADES for the ChCl–lactic acid molar ratio of 1:2. A shift of the O–H stretching in the lactic acid (3395 cm^−1^) and the DES (3301 cm^−1^) can be seen. In addition, a shift of the carbonilic group from 1715 cm^−1^ to 1730 cm^−1^ is present. These shifts are supposed to be present due to the hydrogen interactions between the functional groups.

#### 2.2.2. Density, pH, and Viscosity

Following the measurement method described in Section 3.5, the DES density showed, as expected, a trend inversely proportional to the percentage of added water, with a maximum of 1.1816 mg mL^−1^ and a minimum of 1.1146 mg mL^−1^. In general, a linear density behavior was identified for the percentage of added water; specifically, a linear decrease in the density was observed with the increase of water content in the mixture (a = −0.0015; b = 1.186.; R^2^ = 0.9939). It is well-known that pH control is crucial in many of the applications of DESs. In fact, this parameter can be effective in biochemical reactions (in catalysis) and can influence the extraction yield by modulating the state of charge of the target analytes. It has been shown that the pH of deep eutectic solvents is influenced by the molar ratio of the component and the temperature [24,25]. pH values, measured following the procedure described in Section 3.5, ranged between 2.08 at T = 24 °C and 1.2 at 83 °C. The fundamental equation for the general linear model used was the following:

pH = a + b ∗ T (°C)(1)

The model parameters were a = 2.4492 and b = −0.0149, with R^2^ = 0.9853. The negative values of the slope reflect the rate of pH decrease with increasing temperature, as reported in the literature [24,25].

**Figure 3:**
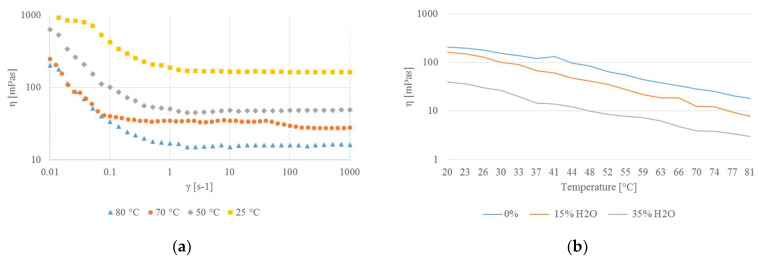
(**a**) Viscosity (η) versus shear rate (γ) change at different temperatures (blue 80 °C, orange 70 °C, grey 50 °C, yellow 25 °C); (**b**) viscosity (η) versus temperature change for different water contents (% *v*/*v*) at g = 1 [s^−1^].

As shown in Figure 3a, the rheological behavior of the ChCl–lactic acid molar ratio 1:2 solvent showed the shear-thinning effect since the viscosity decreased with shear speed, at all tested temperatures. This behavior can be attributed to the breaking of the internal structure of the DES, caused both by the thermal expansion of the structure and the shear effect [26,27,28]. Regarding the change in the viscosity with the percentage of added water in the mixtures, the results shown in Figure 3b confirm the extraction yield trend: the viscosity is inversely proportional to the water content. For example, at the extraction temperature of 80 °C the viscosities measured for 0% (*v*/*v*), 15% (*v*/*v*), and 35% (*v*/*v*) added water were 18.33 mPas, 7.77 mPas, and 3.01 mPas, respectively. This was probably related to the better mixing between the matrix and the extraction solvent that occurs when the water content in the DES is higher. Moreover, we noticed that the percentage decrease of viscosity with increasing temperature (from 20 °C to 80 °C) was around 90% for all the tested water percentages.

### 2.3. Selection of Extraction Parameters and Response Surface Methodology Optimization 

The response surface methodology (RSM) optimization was conducted with the aim of determining the optimal experimental conditions for the total phenolic compounds extracted. Based on our preliminary data and the currently available literature, we selected the following independent variables for the RSM in this study: water content in the DES (15 and 35%); extraction temperature (50, 70, and 80 °C); and solid-to-solvent ratio (1:3.33, 1:5, 1:6.25, 1:10, and 1:25 gmL^−1^).

Maximum temperature was set at 80 °C in order to avoid partial degradation of extracted compounds [29] while maximum percentage of water added was set as 35%. Studies from the literature confirm that the use of different percentages (*v*/*v*) of water in DESs at can be exploited to modulate certain chemical and physical characteristics [30].

Data from previous studies has shown that DES/NADES water systems are stable up to an added water content of around 50% (*v*/*v*) [31]. In addition, Lanjekar et al. used a NADES consisting of ChCl–lactic acid mixed with up to 30% water for the extraction of glycyrrhizic acid from Glycyrrhiza glabra, thus demonstrating its stability, which was confirmed by the unchanged –OH stretching signal between the DES water system and the DES without the addition of water [32]. Higher values are known to cause DES solubilization by breaking up the hydrogen interactions among the components and promoting hydrogen interactions between the water molecules themselves [33]. A previous study showed that a water addition greater than 30% leads to a decrease of genistin, genistein, and apigenin extraction yield from pigeon pea roots, since excessive water can decrease the hydrogen bond strength between the DES and the target compounds [34].

The experimental data acquired in a randomized order (Table 2) were fitted into a second-order polynomial equation (Equation (2)), described in Section 3.8 with the coefficients listed in Table 3. System behavior was represented using three-dimensional response surface plots. Every surface was evaluated by fixing one value for an independent variable and varying the others according to their experimental domains. The total phenolic compounds in the extracts obtained varied from 6.14 (g GAE) 100 g^−1^ of skin to 16.95 (g GAE) 100 g^−1^ of skin, depending on the different levels of the investigated parameters. The lowest extraction yield for all investigated responses was obtained using 15% (*v*/*v*) water, with a temperature of 50 °C and a solid-to-solvent ratio of 1:3.33. Figure 3 shows the response surface plots for the total phenolic compounds concentration for each pair of independent factors.

**Figure 4:**
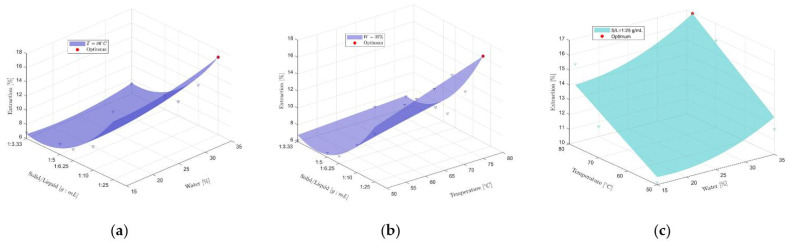
Three-dimensional plots of response surfaces for the effects on the response of (**a**) the solid-to-liquid ratio and percentage of water (g GAE 100 g^−1^ of skin) at three different temperatures; (**b**) the solid-to-liquid ratio and temperature (g GAE 100 g^−1^ of skin) at two different percentages of water; (**c**) the temperature and percentage of water (g GAE 100 g^−1^ of skin) at five different solid-to-liquid ratios.

Figure 4a shows the system response to water content and solid-to-solvent ratio variations at a constant temperature of 80 °C. The increase in response is related to the increase in water percentage and solid-to-solvent ratio. The extraction yield variation between the minimum water content (15% *v*/*v*) and the maximum one (35% *v*/*v*) fixed the solid-to-solvent ratio at 1:25 gmL^−1^, which is about 3%. Figure 4b shows the dependence of the extraction yield on the temperature and the solid-to-solvent ratio, keeping a constant water content of 35% (*v*/*v*). As shown, a higher temperature and higher solid-to-solvent ratio favored extraction. The variation of the extraction yield in response to temperature changes (from 50 °C to 80 °C), at the optimum solid-to-solvent ratio (1:25 gmL^−1^), was about 4.5%. In Figure 4a,b, an increase of 9.44% in extraction yield can be highlighted when varying the solid-to-solvent ratio between the minimum (1:3.33 gmL^−1^) and maximum (1:25 gmL^−1^) values. Figure 4c shows the extraction yield trend in response to varying water percentage and temperature and a fixed solid-to-solvent ratio of 1:25 gmL^−1^. It is clear that higher values for both water content and temperature lead to a better extraction yield.

**Figure 5:**
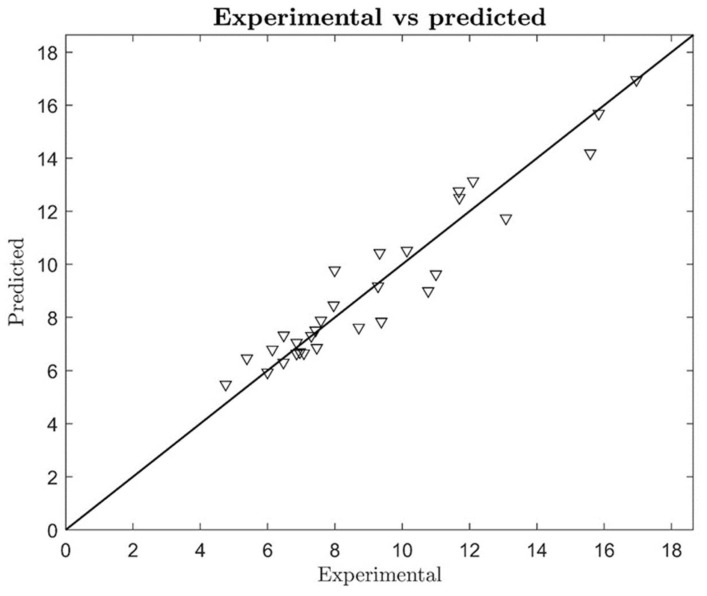
Residues.

To evaluate the correlation between the predicted values of the extraction yield obtained from the polynomial equation and the experimental ones, experimental and predicted values are plotted together in Figure 5. The determination coefficient was calculated as R^2^ = 0.92, indicating a good correlation between predicted and experimental data. The root mean squared error was also evaluated (RMSE = 0.89 g GAE 100 g^−1^ of skin) according to the presented model regression parameters. The optimal predicted point was found at the following conditions: T = 80 °C, W =35%, and SL = 1:25 gmL^−1^, which correspond to a maximum predicted extraction yield of 16.96 g GAE 100 g^−1^ of skin. An additional experimental test was conducted setting T, W, and SL as equal to the optimal model estimation. The extraction yield obtained was 16.80 ± 0.09 g GAE 100 g^−1^ of skin, thus confirming the data.

In order to compare the DES-based extraction with a conventional one, an organic solvent-based UAE was conducted using 0.5 g of skin extracted with 5 mL of EtOH–H_2_O 50% (*v*/*v*) mixtures at room temperature for 30 min. The total concentration of extracted phenolic compounds was 10.22 g GAE 100 g^−1^ of skin. Under optimal conditions, the DES-based system makes it possible to extract in a single step 39% more than the amount extracted by a single-step extraction with EtOH–H_2_O 50% (*v*/*v*) solvent.

### 2.4. HPLC-PDA/MS Analysis of Hazelnut Skin Extract

**Figure 6:**
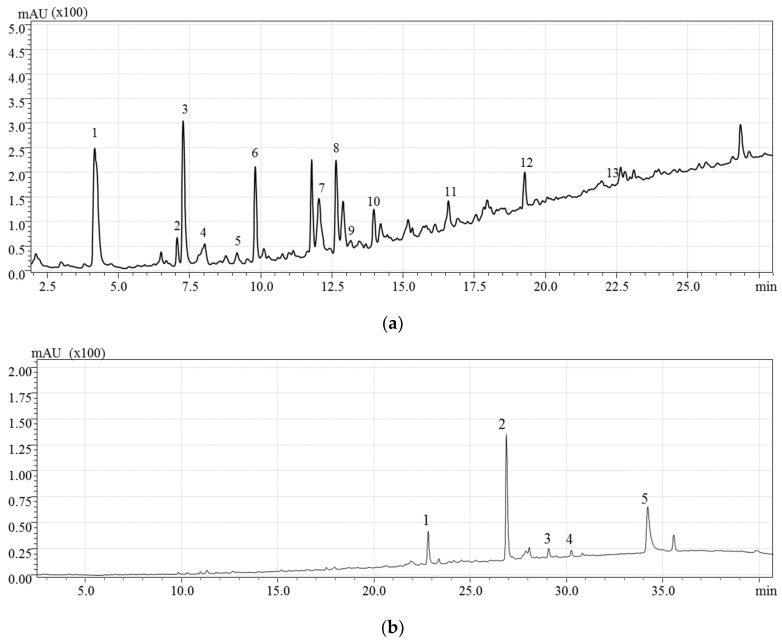
HPLC-PDA chromatogram of hazelnut skin phenolic compounds extract. (**a**) Compounds detected at 280 nm: 1, gallic acid; 2, protocatechuic acid; 3, procyanidin trimer C2; 4, prodelphinidin beta-type dimer; 5, prodelphinidin beta-type dimer; 6, prodelphinidin beta-type dimer; 7, procyanidin beta 1 dimer; 8, (+) catechin; 9, procyanidin beta-type trimer; 10, procianidin beta-type trimer; 11 (−) epicatechin; 12, procyanidin beta-type dimer gallate; 13, epicatechin 3-*O*-gallate. (**b**) Compounds detected at 360 nm: 1, myricetin rhamnoside; 2, quercetin-3-*O*-rhamnoside; 3, ploretin-2-*O*-glucoside; 4, kampferol rhamnoside; 5, quercetin.

Figure 6 shows the HPLC profile, at 280 and 360 nm, of the phenolic compounds extracted using the conventional solvent identified. Table 4 reports the phenolic compounds tentatively identified, including the experimental *m*/*z* values. As shown in Table 4, the identified phenolic compounds were flavan-3-ols and phenolic acids.

The putative identification of the 18 detected compounds was based on the mass-to-charge ratio (*m*/*z*) of the molecular ion, the retention time, and UV data from the literature [1]. Whenever possible, analytical standards were used to confirm both the retention times and the mass-to-charge ratios of the compounds.

Flavan-3-ols (+)-catechin and (−)-epicatechin were distinguished by their different retention times (10.2 min and 14 min, respectively), since they have the same molecular ion *m*/*z* ratio (289 *m*/*z*). These flavan-3-ols were detected in all the hazelnut components (i.e., shell, kernel, and skin) [1,35,36]. Epicatechin 3-*O*-gallate was detected with [M − H]^−^ at *m*/*z* 441, confirming the results obtained by Del Rio et al. (2011) [1]. Among proanthocyanidins, one B-type procyanidin (PC) dimer was identified ([M − H]^−^ at *m*/*z* 577). Three procyanidin trimers were detected with a mass-to-charge ratio of 865 *m*/*z*. Based on the elution time, which precedes that of procyanidin B1, one was tentatively identified as procyanidin trimer C2, while the others were identified as procyanidin beta-type trimers. Within the phenolics acids, protocatechuic acid ([M − H]^−^ at 153 *m*/*z*) and gallic acid ([M − H]^−^ at 169 *m*/*z*) were also identified through comparison with analytical standards and in accordance with data from previous studies (Del Rio et al., 2011 [1], Slatnar et al., 2014 [36], and Yuan et al., 2018 [37]). Three flavonol rhamnoside compounds were identified, these being myricetin rhamnoside ([M − H]^−^ at 463 *m*/*z*), quercetin 3-*O*-rhamnoside ([M − H]^−^ at 447 *m*/*z*), and kaempferol rhamnoside ([M − H]^−^ at 431 *m*/*z*). The same compounds have been previously detected in hazelnut skin [1] and, with the exception of kaempferol rhamnoside, they have also been previously identified in leaves, shells, and kernels of C. avellana L. [35,36,37]. Quercetin ([M − H]^−^ at 301 *m*/*z*) was also detected by comparison with analytical standards. Moreover, this aglycon has been previously identified in hazelnut skin and shell [1,37]. Finally, phloretin 2-*O*-glucoside ([M − H]^−^ at *m*/*z* 435), belonging to the dihydrochalcone subclass, was also identified.

## 3. Materials and Methods

### 3.1. Chemicals

ChCl, triethylene glycol, glycerol, urea, and sucrose were obtained from Sigma-Aldrich (Milan, Italy); sorbitol and glucose were purchased from Fisher Scientific Italia (Milan, Italy); and betaine anhydrous, xylitol, citric acid, ethylene glycol, propylene glycol, 1,6-hexanediol, lactic acid, and malic acid were purchased from Carlo Erba (Milan, Italy). Gallic acid, procyanidin B2 (PB2), procyanidin A2 (PA2), epicatechin, quercetin, kaempferol, methanol (MeOH) (purity 99.9%), water (HPLC-MS grade), acetonitrile (purity 99.9%), and formic acid (purity 95–97%) were obtained from Sigma-Aldrich (Milan, Italy). All the chemicals were of analytical reagent grade and were used without any further purification.

### 3.2. Samples

Hazelnut skins were obtained from a company that uses hazelnuts as a raw material for the preparation of food products. Before the experiments, an aliquot of sample was ground in a coffee grinder (Moulinex Super Junior) and stored at room temperature until use.

### 3.3. Preparation of DESs

Due to its hygroscopicity, ChCl was dried in an oven for one night at 90 °C before preparing the DESs. HBA and HBD were weighed in the established molar ratio and placed in a round-bottom flask. The mixture was heated at 80 °C until a homogeneous liquid was formed. Then, water was added (35% (*v*/*v*)) in order to reduce the viscosity of the DESs. All the selected DESs are summarized in Table 1.

### 3.4. FTIR Spectroscopy

DESs were analyzed by FTIR analysis with a Nicolet 8700 to ensure the formation of the DES. The spectra were acquired at room temperature with six replicates (64 scans for acquisition); the wavenumber measurement range was from 4000 to 400 cm^−1^ with a 4 cm^−1^ resolution.

### 3.5. pH, Density, and Viscosity of DESs

A 0.5 M aqueous solution of DES was prepared for pH measurements and kept at room temperature until use, following Skulcova et al., 2018 [38]. The pH was measured at different temperatures in a range from 25 °C to 80 °C by heating the mixture in a water bath on a heating plate. pH was determined using a digital pH meter from Eutech Instruments^®^ (Toronto, ON, Canada). DES density was measured by weighing a 1 mL flask filled with the DES on an analytical balance from Sartorious (Gottinga, Germany) at room temperature. The dynamic viscosity (η) was measured with an Anton Paar rheometer MCR 302 (Anton Paar, Austria) in a shear rate range between 0.01 and 1000 s^−1^, with a parallel-plate measurement geometry with a gap of 0.100 mm. The viscosity was investigated across a range of temperatures (25 °C to 80 °C) and for three different percentages of water in the DES (0%, 15%, and 35%) in order to correlate these characteristics with the extraction efficiency. All the experimental measurements were carried out in triplicate and the mean values were used.

### 3.6. Ultrasound-Assisted Extraction of Phenolic Compounds with a Conventional Solvent

In order to compare the efficiency of DES-based extraction with a conventional solvent-based one, a method from the literature was chosen [1], with some modifications. First, 0.5 g of sample was extracted using 5 mL of a mixture of EtOH and H_2_O (50:50, *v*/*v*) in a centrifuge tube, and the mixture was placed in an ultrasound bath (Elmasonic S30H, Elma Schmidbauer GmbH, Singen, Germany) at 50 °C, frequency of 37 kHz and heating power of 200 W, for 60 min. Then, the tube was centrifuged at 3214 g for 5 min and the supernatant was filtered in a 0.45 μm syringe filter. The solution was dried under vacuum and reconstituted with 400 μL of MeOH–H_2_O 50:50 (*v*/*v*) mixture. Each extraction was conducted in triplicate and all the extracts were stored at −80 °C until use.

### 3.7. Ultrasound-Assisted Extraction of Phenolic Compounds with DESs

An total of 0.2 g of hazelnut skin was mixed with 5 mL of DES and vortexed for 1 min. The tube was put in an ultrasound bath (Elmasonic S30H, Elma Schmidbauer GmbH, Singen, Germany), frequency of 37 kHz and heating power of 200 W, for 30 min at a temperature of 80 °C with a temperature control of ±2 °C. The mixture was filtered on a Buchner filter and the extract was stored at −80 °C until analysis. Triplicate extractions were conducted for all experiments.

### 3.8. Ultrasound-Assisted Extraction of Phenolic Compounds with DESs

A selection process was performed to select the best experimental conditions for extraction of phenolic compounds. Factors such as water content in the DES, extraction temperature, and the solid-to-solvent ratio were studied.

Two levels were selected for the water content added to the DES mixture (15% and 35%, *v*/*v*), five levels for the solid-to-solvent ratio (gmL^−1^), 1:3.33, 1:5, 1:6.25, 1:10, and 1:25, and three levels for the extraction temperature (50 °C, 70 °C, and 80 °C). For the evaluation of extraction time, preliminary studies showed that after 30 min there was no significant variation in the extracted amount (data not shown) and thus this time period was selected as extraction time. The experiments, conducted by simply matching each of the levels of the factors to each other, are summarized in Table 2. Experimental data were fitted into a second-order polynomial model and regression coefficients were obtained. The polynomial model formula was as follow:

y = a_1_W^2^ + a_2_T^2^ + a_3_SL^2^ + a_4_WT + a_5_ T SL + a_6_ W SL + a_7_ W + a_8_T + a_9_SL + a_10_ (2)

where a_1–10_ are the coefficients of the polynomial model equation obtained by means of a nonlinear regression, while W (water content, % *v*/*v*), T (temperature, °C), and SL (solid-to-solvent ratio, gmL^−1^) represent the independent coded variables and y, the model output, is the extracted amount in g GAE 100 g^−1^ of skin. Each variable was mapped linearly in the interval [–1;1], where −1 corresponded to the variable minimum value and 1 to the variable maximum value. MATLAB^®^ was used as the software for the regression and the response surfaces analysis and visualization. The analysis consisted of evaluating the absolute maximum of the curve, which corresponded to the optimal operating conditions confirmed experimentally.

### 3.9. HPLC-PDA/ESI-MS Analysis of Phenolic Compounds

The analysis was performed using a Shimadzu Prominence LC-20A instrument (Shimadzu, Milan, Italy) equipped with two LC-20 AD XR pumps—a SIL-10ADvp pump and a CTO-20 AC column oven—and a DGU-20 A3 degasser coupled to a SPD-M10Avp PDA detector and a mass spectrometer detector (LCMS-2010, Shimadzu, Tokyo, Japan), equipped with an electrospray (ESI) interface. Shimadzu LCsolution version 3.7 software (Shimadzu, version 3.7) was used to acquire the MS data. The separation of the analytes was carried out using a Core Shell C18 column (150 × 4.6 mm I.D., 2.7 μm d.p.) (Merck KGaA, Darmstadt, Germany). Elution was performed at a constant flow rate of 1 mLmin^−1^ and at a temperature of 40 °C. The mobile phase was (A) 0.1% (*v*/*v*) HCOOH aqueous solution and (B) acetonitrile with 0.1% (*v*/*v*) HCOOH. Polyphenols were separated using the following gradients: 0–40 min 0–30% B, 40–41 min 100% B. The injection volume was 2 μL. Data were acquired using aPDA in the range 200–400 nm and the chromatograms were extracted at 280 and 360 nm. MS chromatograms were acquired in negative ionization mode, using the following parameters: nebulizing gas flow rate (N_2_): 1.5 mL min^−1^; event time: 1 s; mass spectral range: *m*/*z* 100–800; scan speed: 1000 amu/s; detector voltage: 1.5 kV; interface temperature: 250 °C; CDL temperature: 300 °C; heat block temperature: 300 °C; interface voltage: −3.50 kV; Q-array voltage: 0.0 V; Q-array RF: 150.0 V.

### 3.10. Determination of Total Phenolic Compounds Content 

The total phenolic compounds (TPCs) content was determined by oxidation of phenolic compounds using Folin–Ciocalteu’s reagent [39]. Briefly, 20 µL of sample were mixed with 100 µL of Folin–Ciocalteu’s reagent and 1580 µL of EtOH:H_2_O (50% *v*/*v*) mixture and kept in the dark for 10 min. Then, 300 µL of an aqueous solution of Na_2_CO_3_ 0.2 g mL^−1^ was added and put back in the dark for 2 h under continuous stirring. Finally, the mixture was centrifuged for 2 min at 10,621 g and 200 µL of the sample was put in a Greiner microplate. The absorbance was measured with the Infinite M200 PRO Tecan microplate spectrophotometer (Tecan Trading AG, Switzerland) at 765 nm. The concentration of the samples was calculated by interpolating the result in a calibration curve made using gallic acid as an analytical standard in a range from 0 to 2000 µg mL^−1^. Results were expressed as grams of gallic acid equivalent (g GAE) per 100 g^−1^ of skin.

## 4. Conclusions

The extraction of bioactive compounds such as flavan-3-ols and phenolic acids from hazelnut skin was studied with deep eutectic solvents. HPLC-PDA/MS analysis revealed the presence of 18 compounds in the samples. Among the DESs tested, the solvent composed of ChCl as the HBA and lactic acid as the HBD (NADES-2) was selected as the most promising neoteric solvent. This eutectic solvent was successfully employed in a simple and fast extraction procedure supported by UA and optimized with the response surface methodology. The individual optimized factors can be classified according to their contribution to maximizing extraction yield as follows: solid-to-solvent ratio, temperature, and percentage of added water in the DES. Under optimal conditions, this system makes it possible to extract 39% more than the amount extracted with organic solvent-based extraction. Overall, this work contributes to the possibility of developing an efficient and rapid extraction process to recover natural antioxidants from hazelnut byproducts using bio-renewable DESs, as an opportunity to stabilize new food waste recovery strategies based on sustainable solvents.

## Figures and Tables

**Table 1:** List of the DESs and NADESs used in this study.

Abbreviation	HBA	HBD	Molar Ratio
DES-1	ChCl	Urea	1:2
NADES-2	ChCl	Lactic acid	1:2
NADES-3	ChCl	Glucose	2:1
NADES-4	ChCl	Xylitol	1:2
NADES-5	ChCl	Glycerol	1:2
DES-6	ChCl	1,6-Hexanediol	1:7
DES-7	ChCl	Triethylene glycol	1:2
DES-8	ChCl	Ethylene glycol	1:2
DES-9	ChCl	Propylene glycol	1:2
NADES-10	ChCl	Malic acid	1:2
NADES-11	Betaine	Glycerol	1:2
DES-12	Betaine	Ethylene glycol	1:2
DES-13	Betaine	Triethylene glycol	1:2
NADES-14	Betaine	Lactic acid	1:2
NADES-15	Betaine	Malic acid	1:2

**Table 2:** Dataset of the experiment.

Standard Order ^a^	Run Order ^b^	T °C ± 2	Time (min)	%H_2_O (*v*/*v*)	g skin/5 mL
1	9	50	30	15	0.2
2	23	50	30	15	0.5
3	19	50	30	15	0.8
4	3	50	30	15	1
5	26	50	30	15	1.5
6	7	50	30	35	0.2
7	13	50	30	35	0.5
8	17	50	30	35	0.8
9	5	50	30	35	1
10	29	50	30	35	1.5
11	14	70	30	15	0.2
12	8	70	30	15	0.5
13	15	70	30	15	0.8
14	22	70	30	15	1
15	28	70	30	15	1.5
16	25	70	30	35	0.2
17	10	70	30	35	0.5
18	1	70	30	35	0.8
19	20	70	30	35	1
35	12	70	30	35	1.5
21	11	80	30	15	0.2
22	24	80	30	15	0.5
23	18	80	30	15	0.8
24	6	80	30	15	1
25	16	80	30	15	1.5
26	27	80	30	35	0.2
27	4	80	30	35	0.5
28	21	80	30	35	0.8
29	30	80	30	35	1
30	2	80	30	35	1.5

^a^ Not randomized; ^b^ randomized.

**Table 3:** Regression coefficients of predicted quadratic polynomial model.

	Quadratic			Crossproduct			Linear			
Coefficient	a_1_	a_2_	a_3_	a_4_	a_5_	a_6_	a_7_	a_8_	a_9_	a_10_
	0.6394	0	2.8390	0.1928	−0.9318	−0.4731	0.7166	1.0958	−3.3169	6.7465

**Table 4:** Chromatographic data regarding the phenolic compounds identified in hazelnut skin extract by HPLC-PDA/ESI-MS (280 nm and 360 nm).

**λ 280 nm**		
**Number**	**Compound**	**[M − H]^−^ (*m*/*z*)**
1	Gallic acid	169
2	Protocatechuic acid	153
3	Procyanidin trimer C2	865
4	Prodelphinidin beta-type dimer	593
5	Prodelphinidin beta-type dimer	593
6	Prodelphinidin beta-type dimer	593
7	Procyanidin beta 1 dimer	577
8	(+) Catechin	289
9	Procyanidin beta-type trimer	865
10	Procyanidin beta-type trimer	865
11	(−) Epicatechin	289
12	Procyanidin beta-type dimer gallate	729
13	Epicatechin 3-*O*-gallate	441
**λ 360 nm**		
**Number**	**Compound**	**[M − H]^−^ (*m*/*z*)**
1	Myricetin rhamnoside	463
2	Quercetin-3-*O*-rhamnoside	447
3	Ploretin-2-*O*-glucoside	435
4	Kampferol rhamnoside	431
5	Quercetin	301

## Data Availability

Not applicable.

## Sample Availability

Samples of the compounds extracted from hazelnut skin are available from the authors.
